# Re-examining COVID-19 Self-Reported Symptom Tracking Programs in the United States: Updated Framework Synthesis

**DOI:** 10.2196/31271

**Published:** 2021-12-06

**Authors:** Miranda Lynn Janvrin, Jessica Korona-Bailey, Tracey Pérez Koehlmoos

**Affiliations:** 1 The Henry M Jackson Foundation for the Advancement of Military Medicine Bethesda, MD United States; 2 Uniformed Services University of the Health Sciences Bethesda, MD United States

**Keywords:** COVID-19, coronavirus, framework analysis, information resources, monitoring, patient-reported outcome measures, self-reported, surveillance, symptom tracking, synthesis, digital health

## Abstract

**Background:**

Early in the pandemic, in 2020, Koehlmoos et al completed a framework synthesis of currently available self-reported symptom tracking programs for COVID-19. This framework described relevant programs, partners and affiliates, funding, responses, platform, and intended audience, among other considerations.

**Objective:**

This study seeks to update the existing framework with the aim of identifying developments in the landscape and highlighting how programs have adapted to changes in pandemic response.

**Methods:**

Our team developed a framework to collate information on current COVID-19 self-reported symptom tracking programs using the “best-fit” framework synthesis approach. All programs from the previous study were included to document changes. New programs were discovered using a Google search for target keywords. The time frame for the search for programs ranged from March 1, 2021, to May 6, 2021.

**Results:**

We screened 33 programs, of which 8 were included in our final framework synthesis. We identified multiple common data elements, including demographic information such as race, age, gender, and affiliation (all were associated with universities, medical schools, or schools of public health). Dissimilarities included questions regarding vaccination status, vaccine hesitancy, adherence to social distancing, COVID-19 testing, and mental health.

**Conclusions:**

At this time, the future of self-reported symptom tracking for COVID-19 is unclear. Some sources have speculated that COVID-19 may become a yearly occurrence much like the flu, and if so, the data that these programs generate is still valuable. However, it is unclear whether the public will maintain the same level of interest in reporting their symptoms on a regular basis if the prevalence of COVID-19 becomes more common.

## Introduction

Over the past few years, the COVID-19 pandemic has resulted in more than 175 million deaths around the globe, and it has fundamentally changed the lives of millions more, demanding continual innovation and ingenuity. The increasing isolation brought on by the recommended quarantine and social distancing guidelines has led to further reliance on technology for both employment and social interaction [1,2]. Capitalizing on this, researchers in the field of participatory epidemiology developed programs to track individuals’ symptoms and map trends within communities.

These developments were further spurred by the delay in traditional epidemiological surveillance. Although epidemiological surveillance is fundamental in coordinating strategies for detection and prevention [3], in the early stages of the COVID-19 pandemic, there were many instances where testing was not available or otherwise inaccessible [4]. It was postulated that this delay could be partially assuaged through an uptake in crowdsourced participatory surveillance efforts, based on the successful monitoring of yearly influenza outbreaks [5,6]. Although outbreaks have considerably declined since the advent of the COVID-19 vaccine, the application of COVID-19 symptom tracking technology to the pandemic has afforded researchers ample data both on virus spread and its impact on the individual.

To shed light on these important efforts in the United States, early in the pandemic, in 2020, Koehlmoos et al [7] completed a framework synthesis of currently available self-reported symptom tracking programs for COVID-19—the disease caused by a specific novel coronavirus. They sought to identify programs in the United States that remotely tracked daily symptom fluctuations among participants and mapped disease spread at a community level. This framework described the programs, partners and affiliates, funding, responses, platform, and intended audience, among other considerations. Ideally, this synthesis would raise awareness of programs while identifying gaps and overlaps [7]. This study seeks to update Koehlmoos et al’s framework, with the aim of identifying developments in the landscape and highlighting how programs have adapted to changes in pandemic response.

## Methods

### Study Design

Our team developed a framework to collate information on current COVID-19 self-reported symptom tracking programs using the “best-fit” framework synthesis approach [8]. The innovative best-fit framework was chosen for its strength, utility, and appropriateness in drawing conclusions for an evolving subject, as it does not require a further process of interpretation by policy makers and practitioners in order for them to inform practice [9]. Information on each program was collected and organized into a table for ease of comparison.

### Target Population

The original framework synthesis served to identify programs tracking symptoms associated with COVID-19 in the US population. The updated version includes all programs identified in the prior synthesis, as well as sought to include any programs implemented thereafter. Inclusion and exclusion criteria are described below.

#### Inclusion Criteria

Programs that aim to capture and geographically collate self-reported symptoms associated with COVID-19 that are available for use in the United States were included for the analysis. For this synthesis, a symptom tracking program is defined as a program that allows individuals to report COVID-19 symptoms, in an effort to identify geographic areas with emerging disease or changes in disease progression.

#### Exclusion Criteria

Programs that do not track specific symptoms for COVID-19, those that were symptom checkers intended for individual use only, or those that did not target the US population were excluded from the analysis.

### Program Identification

All programs from the previous synthesis were included to document changes, if any. New programs were discovered using a Google search for keywords (eg, “symptom trackers covid,” “symptom trackers coronavirus,” “symptom tracking covid,” “symptom tracking coronavirus,” “daily symptom tracking covid,” “daily symptom tracking coronavirus,” “self-reporting covid,” “self-reporting coronavirus”). The time frame for the search for programs ranged from March 1, 2021, to May 6, 2021. This 2-month timeline was selected to reflect the state of COVID-19 symptom tracking programs 1 year after our previous synthesis.

### Screening Method

Reviewers (JK, MLJ, and TPK) screened programs to determine whether inclusion criteria were met, and if so, they extracted data from program websites using a standardized form. In the case of conflict between reviewers, final determinations were made by TPK. To gather information that may not be available via program websites, program managers were contacted via email.

### Synthesis Method

Data related to program characteristics were extracted from all included programs and organized into a table using the tool developed in the previous synthesis to guide data collection and build the framework for analysis. Data were then synthesized to form meaningful statements about the programs.

## Results

We identified 33 programs in total, 8 (24%) of which met the inclusion criteria. All 6 programs identified in the last synthesis were included to document changes, if any. Notably, 2 of those 6 programs had been rebranded since our original synthesis, with COVIDNearYou now known as Outbreaks Near Me and COVID Symptom Tracker now known as COVID Symptom Study. Two newly identified programs, COVIDSymptom and COVID Control, met the inclusion criteria. Thus, information was gathered from all 8 eligible programs (ie, BeatCOVID19Now, COVIDcast, COVID Control, COVIDSymptom, COVID Symptom Study, HelpBeatCOVID19, HowWeFeel, and Outbreaks Near Me; see Table 1). In all, 25 programs were excluded as they did not meet the inclusion criteria (Multimedia Appendix 1). Of the original 6 programs, BeatCOVID19Now has since been terminated, reportedly due to control of disease spread in its home location (Australia), resulting in lack of interest in continuing efforts in the United States and other international settings. Hence, BeatCOVID19Now is not included in the tabulated data.

**Table 1:** Overview of self-reported symptom tracker programs.

Characteristic	Program name						
	COVIDcast	COVID Control	COVIDSymptom	COVID Symptom Study^a^	HelpBeatCOVID19	HowWeFeel	Outbreaks Near Me^b^
Host institution and partners	Carnegie Mellon University Delphi Research Group Facebook COVID Symptom Study^c^ Outbreaks Near Mec	John Hopkins Bloomberg School of Public Health John Hopkins School of Medicine John Hopkins Whiting School of Engineering John Hopkins Medicine Technology Innovation Center University of Vermont Medical Center Capitol Technology University ITC Infotech Digital Experience	University of Michigan Kirusa NJ Tech Council NJ Business & Industry Association Walk-In Medical Urgent Care Sills, Cummis & Gross Decagon Strand SpectraMEDI	Harvard T.H. Chan School of Public Health Massachusetts General Hospital King's College London Stanford University School of Medicine ZOE COVIDcast^c^ Outbreaks Near Mec Agricultural Health Study^c^ American Cancer Society^c^ Aspree XT^c^ Black Women’s Health Study^c^ California Teachers Study^c^ Cancer Prevention Study–3^c^ Dr. Susan Lovec Foundation Gulf Study^c^ Gutsc The Multiethnic Cohort Study^c^ The Sister Study^c^ Stand Up to Cancer^c^ University of Texas School of Public Health^c^	University of Alabama Alabama Department of Public Health	Harvard T.H Chan School of Public Health MIT^d^ IQSS^e^ McGovern Institute Howard Hughes Medical Institute Weizmann Institute of Science Pinterest Feeding America Alex’s Lemonade Stand Chartio Bill & Melinda Gates Foundation Stanford University^c^ University of Pennsylvania^c^	Harvard Medical School Boston Children’s Hospital Ending Pandemics Google Centers for Disease Control COVID Symptom Study^c^ COVIDcast^c^
Location	Pittsburgh, PA	Baltimore, MD	Ann Arbor, MI	Boston, MA	Birmingham, AL	Boston, MA	Boston, MA
Funding sources	None	White and Case ITC Infotech	New Jersey Technology Control Kirusa	Mass General Wellcome Trust (UK)	University of Alabama	Bill and Melinda Gates Foundation Crowdsourcing	Ending Pandemics Crowdsourcing AtScale^c^ AWS^c,f^ BlazeMeter^c^ Cloudflare^c^ Datadog^c^ MongoDB^c^ SurveyMonkey^c^ TechSoup^c^
Intended participants (age in years)	US residents (18+)	US residents (13+)	Worldwide (18+)	US residents (18+) Participants from other internal studies, including RCTs^g^	US residents (18+); particular focus on Alabama and neighboring states	US residents (18+)	United States, Canada, and Mexico residents (18+)
Date symptom tracker was initiated	April 2020	April 2020	June 2020	April 2020	Not available	April 2020	March 2020
Number of responses to date^c^	19,989,000 (+17,415,000)^c^	215,000	—^h^	4,651,000 (+4,553,000)^c^	101,000 (+44,000)^c^	—	5,867,000 (+5,813,000)^c^
Mechanism of recruiting participants or platform	Survey via Facebook	Apple App Store Google Play Store	Apple App Store Google Play Store	Apple App Store Google Play Store	Web browser	Apple App Store Google Play Store	Web browser
Follow-up	Daily survey prompts via Facebook^c^	Daily phone notifications	Daily phone notifications	Daily phone notifications	SMS notifications every three days	Customizable phone notifications^c^	Daily SMS notifications^c^
Frequency of reporting	Daily	Daily	—	Live Data	Live Data	Daily	Weekly
Availability of summary tables for external synthesis or utilization	Yes	Yes	Yes	Yes	No	No	Yes
Intended audience for the product	Public at large State and local public health officials US policy makers Health care providers Health care systems CDCi and national public health organizations^c^ Researchers^c^	Public at large State and local health officials Researchers	Public at large Health care providers Researchers	Public at large Participants of internal studies CDC and national public health organizations^c^ Health care providers^c^ Health care systems^c^ Researchers^c^ State and local public health officials^c^ US policy makers^c^	Public at large Neighboring States State and local health officials Local policy makers	Public at large State and local public health officials Health care providers Health care systems Researchers State and local public health professionals CDC and national public health organizations	Public at large CDC and national public health organizations State and local public health officials Researchers Health care providers Health care systems US policy makers
Publicly available data privacy statement	Yes	Yes	Yes	Yes	Yes	Yes	Yes

^a^Formerly known as COVID Symptom Tracker.

^b^Formerly known as COVIDNearYou.

^c^Indicates changes made since the previous synthesis.

^d^MIT: Massachusetts Institute of Technology.

^e^IQSS: Institute for Quantitative Social Science.

^f^AWS: Amazon Web Services.

^g^RCT: randomized controlled trial.

^h^Not available.

^i^CDC: Centers of Disease Control and Prevention.

All 6 of the previously reported programs have maintained their affiliation with a university, school of medicine, or school of public health. The 2 newly added programs are also affiliated with universities, further emphasizing the importance of academic institutions in studying the spread of COVID-19 in the United States. Three programs continue to be based in Boston, MA (ie, COVID Symptom Tracker, HowWeFeel, and Outbreaks Near Me), with another affiliated through partnerships (COVIDcast). However, the 2 new programs are based in Ann Arbor, MI and Baltimore, MD.

All of the programs continued to receive responses through electronic reporting mechanisms, with 2 programs utilizing a web browser-based approach (ie, Outbreaks Near Me and HelpBeatCOVID19). Four programs, including both the newly added programs, use apps for both Apple and Android devices; these include COVID Control, COVIDSymptom, COVID Symptom Study, and HowWeFeel. One program (COVIDcast) uses social media as a platform for their survey.

Upon reviewing the data elements being collected across the programs (Table 2), it appears that some of the trackers have been revised to ask more timely questions, including those covering topics like vaccination (eg, COVIDcast, COVID Symptom Study, HowWeFeel, Outbreaks Near Me), vaccine hesitancy (eg, COVIDcast and HowWeFeel), adherence to social distancing recommendations put forth by the Centers for Disease Control and Prevention (CDC; eg, COVIDcast and HowWeFeel), and in-person schooling for children K-12 (eg, HowWeFeel). Notably, COVIDcast asks about mask usage. The remaining programs (COVID Control, COVIDSymptom, HelpBeatCOVID19, and Outbreaks Near Me) continue to focus their efforts only on elements such as ongoing symptoms, testing status, and demographic information. Interestingly, COVIDcast and COVID Symptom Tracker have limited or removed much of the demographic data that they collect.

**Table 2:** Data elements across programs.

Question	COVIDcast	COVID Control	COVID Symptom	COVID Symptom Study	HelpBeatCOVID19	HowWeFeel	Outbreaks Near Me
Are you completing the survey on behalf of someone else?				✓	✓		
Age		✓	✓		✓	✓	✓
Gender		✓	✓		✓	✓	✓
Zip code		✓			✓	✓	✓
Race or ethnicity				✓	✓	✓	
Blood group				✓			
Symptoms at time of check-in		✓	✓	✓		✓	
Symptoms within the last 24 hours	✓						
Symptoms over the past 7 days					✓		✓
Date of symptom onset							✓
Do others in your household have similar symptoms to those you reported?	✓				✓	✓	
Temperature at the time of check-in		✓					
Highest temperature over symptom duration			✓	✓	✓	✓	✓
Hours of sleep previous night						✓	
Have you been tested for COVID-19?		✓	✓	✓	✓		
Type of testing or care sought due to symptoms						✓	✓
What type of medical test did you receive							✓
Results of testing		✓	✓	✓	✓		✓
In the past 14 days, did you want a COVID-19 test but did not receive one							✓
How long after you started feeling ill did you see a health professional?							✓
What prescription, if any, did you receive for your illness?							✓
Have you received the COVID-19 vaccine?	✓			✓		✓	✓
Would you accept a COVID-19 vaccine if offered?	✓			✓			
If a safe, effective coronavirus vaccine were available, how likely would you be to get vaccinated?						✓	
What is the main reason you got the COVID-19 vaccine?						✓	
What is the main reason you did not get the COVID-19 vaccine?						✓	
Do you know anyone who has received a COVID-19 vaccine?						✓	
If you have a child under 18, how likely are you to get your child vaccinated?						✓	
Are you experiencing any symptoms near the injection site?				✓			
Preexisting conditions			✓	✓	✓	✓	
Obesity					✓	✓	
Are you pregnant?					✓	✓	
Are you/have you ever been a smoker			✓		✓	✓	
Have you received the flu vaccine?							✓
Number of people in the household					✓	✓	
Are you a parent?						✓	
Number of children in the household						✓	
Do any children in your household (pre-K through grade 12) go to full-time in-person classes?						✓	
Has anyone in your household been diagnosed with COVID-19?					✓		
Type of domicile					✓		
Have you left your home in the past 24 hours?						✓	
Reason for leaving home						✓	
What protective measures did you take when you left home?						✓	
In the past 7 days, did you wear a mask most or all of the time in public?	✓						
In the past 7 days, when you were in public places where social distancing is not possible, did most or all other people wear masks?	✓						
In the past 24 hours, did you spend time indoors with someone who isn’t currently staying with you?	✓						
In the past 24 hours, did you attend an indoor event with more than 10 people?	✓						
In the past 24 hours, did you go to an indoor market, grocery store, or pharmacy?	✓					✓	
In the past 24 hours, did you have a meal or drink indoors at a bar, restaurant, or cafe?	✓					✓	
In the past 24 hours, did you use public transit?	✓						
In the past 7 days, have you traveled outside of your state?	✓						
Have you been in contact with anyone diagnosed with COVID-19?					✓	✓	
Do you personally know someone in your local community who has COVID-like symptoms?	✓						
Does your job require you to leave your home and go to another place to work where you come in contact with public?	✓				✓		
How much of the following feelings (tired, calm, happy, angry, sad, thoughtful, optimistic, anxious, lonely, grateful, hopeful, stressed) have you felt so far today?	✓					✓	
Do you feel very or somewhat worried about becoming seriously ill from COVID-19?	✓						
Do you feel very or somewhat worried about your household’s finances for the next month?	✓						
Health insurance status					✓		
If you needed to get to a hospital or testing center, how would you get there?					✓		
Can you afford any payment or co-payment required for services?					✓		

All 7 currently active programs collect information regarding symptoms, although the time period varies, with the majority (4/7, 57%), asking participants to report current symptoms at the time of check-in (ie, COVID Control, COVIDSymptom, COVID Symptom Tracker, and HowWeFeel), 2 (29%) asking about symptoms experienced over the past week (ie, HelpBeatCOVID19 and Outbreaks Near Me), and only 1 (14%) asking about symptoms experienced over the past 24 hours (ie, COVIDcast). Except for COVIDcast, all other programs ask symptomatic participants who report fever for their temperature, with COVID Control uniquely asking participants who report to have experienced no symptoms. All of these programs (excluding COVIDcast) also ask participants whether they have undergone testing, and 5 programs (ie, COVID Control, COVIDSymptom, COVID Symptom Tracker, HelpBeatCOVID19, Outbreaks Near Me) ask about the results.

COVID Control and COVIDSymptom limited their questions to symptoms, testing, and demographic data, whereas other programs had unique questions, indicating their areas of focus. COVIDcast tailored its questions to current issues, including vaccination status, adherence to social distancing guidelines, mask usage, use of public transit, travel, and mental health concerns. COVID Symptom Study also asked participants about vaccination status, as well as vaccine hesitancy, vaccine side effects, and it was the only program to ask participants about their blood group. HelpBeatCOVID19 asked participants about their household, health insurance status, and financial insecurity. HowWeFeel asked questions on vaccination status; vaccine hesitancy, including asking participants why they have or have not been vaccinated and whether they would vaccinate their children; adherence to social distancing guidelines; and mental health concerns. Finally, Outbreaks Near Me asks participants in-depth questions about testing, including what kind of test participants receive, their diagnosis, and any medications they are prescribed.

## Discussion

### Principal Findings

Over the course of the past year, symptom tracking programs have been exceedingly useful for predictive modeling and population research throughout the COVID-19 pandemic [10]. The data from COVID Symptom Study, for example, has already been used to unearth COVID-19–related trends, even revealing 6 distinct “types” of COVID-19 emerging among their participants. The importance of their contributions were highlighted in the *New England Journal of Medicine* article “Putting the Public Back in Public Health—Surveying Symptoms of Covid-19” [10]. This framework update shows the increase in responses, collaboration, and evolution of questionnaires between programs exemplifying their use by the public health community.

Many symptom checking programs (not included in this framework), defined—for the purposes of this study—as programs that serve to indicate whether the participant likely should or should not seek medical attention without aiming to record symptoms over time, have emerged and with good reason [11]. These programs are critical in deterring those without serious symptoms from seeking emergency care. However, the number of symptom tracking programs has not correspondingly increased. Only 2 additional symptom tracking programs were identified, which indicates either the demand for these programs is significantly lower than symptom checking programs or that the current programs are doing well to serve this niche. It is also possible that both reasons are true.

Reported responses to each of the original 6 programs substantially increased. The lowest number of responses, defined as unique symptom entries by an individual, were received for HelpBeatCOVID19—recorded at 101,000, an increase of 44,000. The remaining 3 programs with reported responses also showed exponential increase in the number of responses. For instance, COVIDcast had 19,989,000 responses, an increase of 17,415,000—the highest of all programs. COVID Symptom Study recorded 4,651,000 responses, an increase by 4,553,000. Outbreaks Near Me recorded 5,867,000 responses, an increase of 5,813,000. In the original framework, two-thirds of the programs had less than 100,000 responses. Among the newly added programs, COVID Control had 215,000 responses. Although only half of the previous programs focused on user retention through notifications (ie, COVID Symptom Study, Outbreaks Near Me, and HelpBeatCOVID19), we found that all programs now prompted previous participants on a daily or semiregular basis through either phone or social media notifications or SMS text messages.

Despite the addition of 2 new programs to the framework, 1 program concluded during the past year. BeatCOVID19Now, based in Australia, concluded and terminated its website in early 2021. Notably, it was the only program in the original synthesis that served international participants and was founded by the same researchers who developed the Influenza Intensity and Impact Questionnaire (fluiiQ) [12]. The discontinuation of BeatCOVID19Now also raises the question of the longevity of the other programs—like BeatCOVID19Now, Outbreaks Near Me was developed based on existing infrastructure, namely Flu Near You, which performed a similar function for yearly influenza outbreaks. Therefore, discontinuation of BeatCOVID19Now poses the question of whether other programs may be discontinued in the near future.

Collaboration between programs also seems to be auspicious for growth and user retention. Three of the programs (ie, COVIDcast, COVID Symptom Tracker, and Outbreaks Near Me) have begun collaborating. Lack of collaboration was a chief concern in the first iteration of our framework synthesis, with the concern being that it would lead to unnecessary duplication and a division of the pool of potential participants, and ultimately, all of the programs sought to share this important data with those who could best utilize it. Although these concerns cannot be completely assuaged without further collaboration among groups, initiating collaboration among the 3 programs with the highest response rates is a step forward.

A notable development was the continued change in data elements observed by the majority of programs. Elements such as symptom status and potential contact continue to be necessary; however, seeing programs adapt to changes in pandemic response by adding elements concerning vaccination, vaccine hesitancy, social distancing, and mental health indicates what could be the next direction for these programs.

### Limitations

As with the first iteration of this study, several limitations must be acknowledged. First, our analysis was limited to English-language programs; therefore, we may have missed nuances of data collection that are more important to non–English-speaking residents. Second, although the speed of framework analysis enables rapid evaluation of commonalities, it does not provide the in-depth rigor of a full systematic review. Third, our collected data evaluated differences in the number of responses to each program, but it only pertained to what data was collected, how that data is being collected, and what groups created and funded each program. Analyzing the effectiveness, market penetration, or user demographics of evaluated programs is beyond the scope of this study. This kind of analysis, if undertaken in a future study, may provide insight into the question of potential longevity posed by this framework. Fourth, we recognize that program participation is limited to only those who have access to the internet or cellular phone service, creating an unintended disparity among respondents based on their access to and utilization of technology. Therefore, the underlying reasons for the difference in response rate remain beyond the scope of this study. Last, this synthesis neither provides critical appraisal of programs nor evaluates programs for effectiveness.

### Conclusions

At this time, the future of self-reported symptom tracking for COVID-19 and for these programs, is unclear. Some sources have speculated that COVID-19 may become a yearly occurrence much like the flu, and if so, the data that these programs generate is still valuable [13]. However, it is unclear whether the public will maintain the same level of interest in reporting their symptoms on a regular basis if the prevalence of COVID-19 becomes more common, and, for programs like COVIDcast, whether the platform where the survey is hosted will continue to support them. The aforementioned conclusion of BeatCOVID19Now represents a foreboding that could be lying ahead—that lack of interest after virus control through the roll-out of rapid and widely available testing and successful vaccination could lead to a lack of interest among participants, and ultimately discontinuation; however, as of May 2021, these 7 programs have not shown any signs of waning.

## Appendix

Multimedia Appendix 1 Programs excluded from the study.

## Abbreviations

DoD: Department of Defense
fluiiQ: Influenza Intensity and Impact Questionnaire
HJF: Henry M Jackson Foundation for the Advancement of Military Medicine, Inc
USUH: Uniformed Services University of the Health Sciences
