# Supplementary figures and images for: Re-examining COVID-19 Self-Reported Symptom Tracking Programs in the United States: Updated Framework Synthesis
Source: JMIR Form Res. 2021 Dec 6;5(12):e31271. doi: 10.2196/31271 (PMC8651180; doi:10.2196/31271)

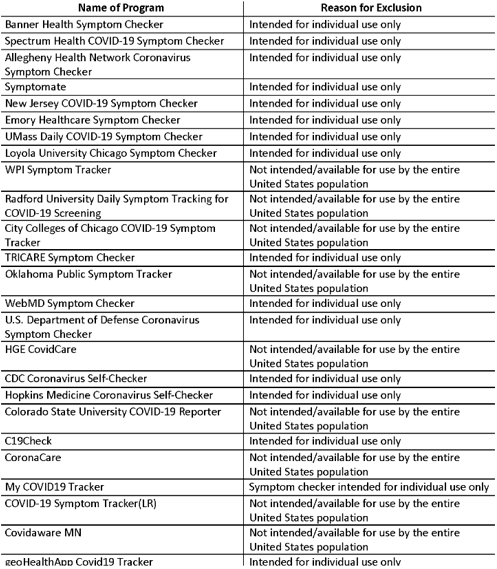

Supplement: Multimedia Appendix 1 [file formative_v5i12e31271_app1.png]
